# Cerebrospinal fluid ctDNA and metabolites are informative biomarkers for the evaluation of CNS germ cell tumors

**DOI:** 10.1038/s41598-020-71161-0

**Published:** 2020-08-31

**Authors:** Takeshi Takayasu, Mauli Shah, Antonio Dono, Yuanqing Yan, Roshan Borkar, Nagireddy Putluri, Jay-Jiguang Zhu, Seiji Hama, Fumiyuki Yamasaki, Hidetoshi Tahara, Kazuhiko Sugiyama, Kaoru Kurisu, Yoshua Esquenazi, Leomar Y. Ballester

**Affiliations:** 1 Department of Pathology and Laboratory Medicine, Molecular Genetic Pathology and Neuropathology, The University of Texas Health Science Center, 6431 Fannin St., MSB 2.136, Houston, TX 77030 USA; 2 Department of Neurosurgery, Graduate School of Biomedical and Health Sciences, Hiroshima University, 1-2-3, Kasumi, Minami-ward, Hiroshima City, Hiroshima 734-8551 Japan; 3 Vivian L. Smith Department of Neurosurgery, UTHealth McGovern Medical School, the University of Texas Health Science Center, Houston, TX USA; 4 Metabolomics Core, Alkek Center for Molecular Discovery, Baylor College of Medicine, Houston, TX USA; 5 Memorial Hermann Hospital-TMC, Houston, TX USA; 6 Department of Cellular and Molecular Biology, Graduate School of Biomedical and Health Sciences, Hiroshima University, Hiroshima, Japan; 7 Department of Clinical Oncology and Neuro-Oncology Program, Hiroshima University Hospital, Hiroshima City, Hiroshima Japan; 8 Center for Precision Health, School of Biomedical Informatics, The University of Texas Health Science Center, Houston, USA

**Keywords:** CNS cancer, Germ cell tumours, Cancer metabolism, Oncogenes

## Abstract

Serum and cerebrospinal fluid (CSF) levels of α-fetoprotein and β-subunit of human chorionic gonadotropin are used as biomarkers for the management of central nervous system (CNS) germ cell tumors (GCTs). However, additional discriminating biomarkers are required. Especially, biomarkers to differentiate non-germinomatous germ cell tumors (NGGCTs) from germinomas are critical, as these have a distinct prognosis. We investigated CSF samples from 12 patients with CNS-GCT patients (8 germinomas and 4 NGGCTs). We analyzed circulating tumor DNA (ctDNA) in CSF to detect mutated genes. We also used liquid chromatography-mass spectrometry to characterize metabolites in CSF. We detected *KIT* and/or *NRAS* mutation, known as frequently mutated genes in GCTs, in 3/12 (25%) patients. We also found significant differences in the abundance of 15 metabolites between control and GCT, with unsupervised hierarchical clustering analysis. Metabolites related to the TCA cycle were increased in GCTs. Urea, ornithine, and short-chain acylcarnitines were decreased in GCTs. Moreover, we also detected several metabolites (e.g., betaine, guanidine acetic acid, and 2-aminoheptanoic acid) that displayed significant differences in abundance in patients with germinomas and NGGCTs. Our results suggest that ctDNA and metabolites in CSF can serve as novel biomarkers for CNS-GCTs and can be useful to differentiate germinomas from NGGCTs.

## Introduction

Germ cell tumors (GCTs) in the central nervous system (CNS) are rare comprising less than 3% of primary brain tumors^1–3^. The incidence is different among races, being higher in Asians (0.136–0.179/100,000 people) than in Whites (0.078/100,000 people)^1,2^. GCTs occur in higher proportion in male patients, with a male: female ratio greater than 3:1^1,2^. GCTs in the CNS predominantly occur in children and adolescents and usually occur in midline structures, typically in the pineal region, third ventricular region, and neurohypophysis. Germinoma is the most common type of GCTs and it is highly radiosensitive. The 5-year survival rate of germinoma exceeds 99%, according to a recent study^3^. In contrast, non-germinomatous GCTs (NGGCTs) require intense chemotherapy, craniospinal irradiation, and sometimes salvage surgical resection^4,5^, and have an unfavorable prognosis with a 5-year survival rate of 71.4–87.3%^3^. Thus, distinguishing between these tumor types is essential to determine the appropriate treatment strategy.

Serum and cerebrospinal fluid (CSF) levels of α-fetoprotein (AFP) and β-subunit of human chorionic gonadotropin (β-HCG), and placental alkaline phosphatase (PLAP) levels in CSF are used as biomarkers for current clinical management of CNS GCTs^4–6^ and in some cases, initial treatment is based on the levels of AFP, β-HCG, and PLAP without tissue confirmation. The pineal region is one of the most frequent sites in which GCTs occur and they often cause non-communicating hydrocephalus.

Intraventricular endoscopy is a feasible and minimally invasive technique, allowing both, access to the lesion for histological analysis as well as CSF diversion via endoscopic third ventriculostomy (ETV). However, biopsied samples for histological analysis, particularly from neuro-endoscopy are often small, representing only a partial region of the tumor, which can lead to inaccuracies in histologic diagnosis due to sampling bias^7,8^. Histologically, GCTs are classified as pure germinoma or non-germinomatous germ cell tumors (NGGCT). However, NGGCT can contain germinoma components, which makes accurate histological diagnosis on small biopsies challenging, and in some cases, inaccurate. Moreover, some patients undergo treatment based on the levels of AFP and β-HCG, without biopsy or surgical resection. Therefore, additional diagnostic biomarkers for GCTs are of critical importance in neuro-oncology. Even though germinomas and NGGCTs are managed with chemotherapy and radiotherapy (not with surgery), therapeutic regimens such as radiation dose, are different and germinomas respond better to treatment^4,5^. Therefore, the distinction between germinoma and NGGCTs is crucial in providing patients with the optimal treatment.

In recent years, CSF has been shown to be a relevant source of informative biomarkers for CNS tumors^9–13^. We have previously reported on the analysis of circulating tumor DNA (ctDNA) and metabolites in the CSF of patients with lymphoma, gliomas, and metastatic CNS tumors^14–16^. In this study, we analyzed both ctDNA and metabolites in CSF from CNS GCT patients, with the aim of identifying biomarkers that could potentially be utilized in clinical practice.

## Results

### AFP and β-HCG levels

**Figure 1:**
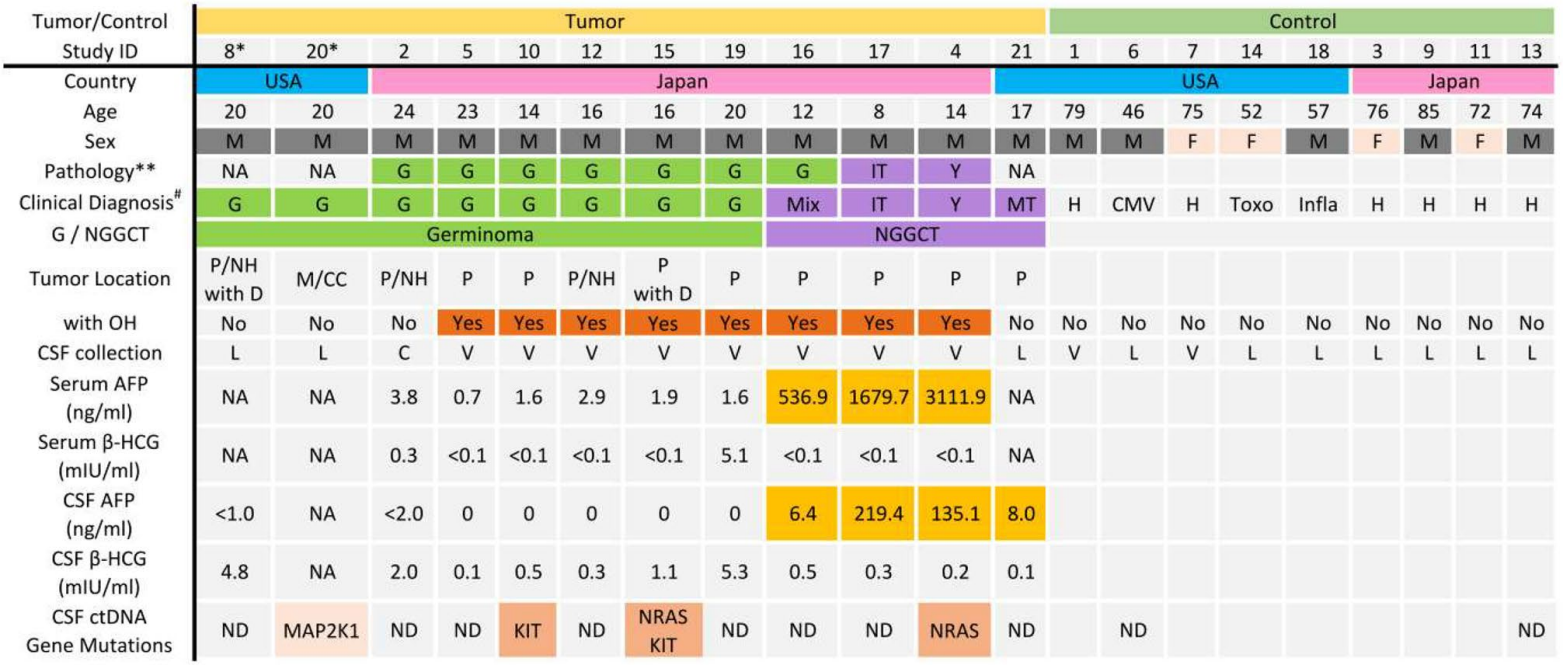
Patient demographics, biomarker levels in GCT patients, and detected mutations in CSF ctDNA are shown. ** represents the histological diagnosis of each patient. # represents the final diagnosis based on the histological report, imaging diagnosis, and biomarkers. *: Case #20 was a recurrence of case #8. *M* male, *F* female, *G* germinoma, *MT* mature teratoma, *Y* yolk-sac tumor, *Mix* mixed germ cell tumor, *IT* immature teratoma, *H* normal pressure hydrocephalus, *CMV* cytomegalovirus infection, *Toxo* toxoplasmosis, *Infla* granulomatous inflammation, *NGGGCT* non-germinomatous germ cell tumor, *P* pineal, *NH* neurohypophysis, *D* dissemination, *M* medulla, *CC* corpus callosum, *OH* obstructive hydrocephalus, *L* lumbar puncture, *C* cistern, *V* ventricle, *ND* not detected.

The biomarker levels of patients are summarized in Fig. 1. All patients with a histologic diagnosis of germinoma, but one, had low CSF AFP level of less than 2.0 ng/ml. One patient’s pathology report indicated germinoma (case #16), but his AFP levels in CSF and serum were 6.4 ng/ml and 536.9 ng/ml, respectively. Given the elevated AFP levels, it was considered that his tumor must have contained teratoma or embryonal tumor components, and it was decided to treat the patient with intensive chemotherapy and radiation. Other NGGCT patients had increased AFP levels in both CSF and serum, which were consistent with their histologic diagnosis. No patients from UTHealth/MHH underwent surgery for tumor resection, and the diagnosis was based on imaging and biomarker levels in CSF.

### Detection of mutations in ctDNA from CSF

We purified CSF ctDNA from 12 samples with CNS GCTs and 9 samples from control patients. There was a limited volume of CSF available and on average 1.71 ml of CSF were used to extract ctDNA. Among tumors, the median concentration and the median amount of extracted cell-free DNA for library preparation were 0.494 ng/μl (range 0.216–45.2) and 5.13 ng (range 2.25–20.0), respectively. Only two samples reached the manufacturer’s recommended amount of 20 ng (Table 1). We detected several gene mutations in CSF-ctDNA from intracranial GCT patients. *KIT*, *NRAS*, and *MAP2K1* (Fig. 1, Table 1) mutations were found in 4 of 12 patients. The mutant allele fraction (MAF) ranged from 1.63 to 40.3%. No mutations were detected in the remaining 8 patients with GCTs or any of the control patients.

**Table 1:** Extracted cfDNA amount and detected genetic alterations.

ID	Tumor/control	Clinical diagnosis	CSF volume for ctDNA extraction (ml)	cfDNA concentration (ng/μL)	Input cfDNA (ng)	Genetic alterations	AF%
06	Control	CMV	1.28	17.5	20.00	ND	
13	Control	NPH	2.88	0.876	9.11	ND	
08	Tumor	Germinoma	2.88	0.684	7.11	ND	
20	Tumor	Recurrent germinoma	1.78	0.556	5.78	MAP2K1 p.F129L	1.6
21	Tumor	Mature teratoma	2.28	45.2	20.00	ND	
02	Tumor	Germinoma	2.88	2.52	20.00	ND	
04	Tumor	Yolk-sac tumor	1.38	0.302	3.14	NRAS p.Q61R	34.6
05	Tumor	Germinoma	1.38	0.266	2.77	ND	
10	Tumor	Germinoma	1.38	0.466	4.85	KIT p.D820G	18.7
12	Tumor	GERMINOMA	0.88	0.264	2.75	ND	
15	Tumor	Germinoma	1.38	0.536	5.57	NRAS p.G12S	40.3
						KIT p.N822Y	32.3
16	Tumor	Germinoma	1.38	0.216	2.25	ND	
17	Tumor	Immature teratoma	1.38	0.342	3.56	ND	
19	Tumor	Germinoma	0.38	0.518	5.39	ND	

*CMV* cytomegalovirus infection, *NPH* normal pressure hydrocephalus, *AF* allele frequency, *ND* not detected.

### Comparison of metabolites in the CSF between germ cell tumors and controls

**Figure 2:**
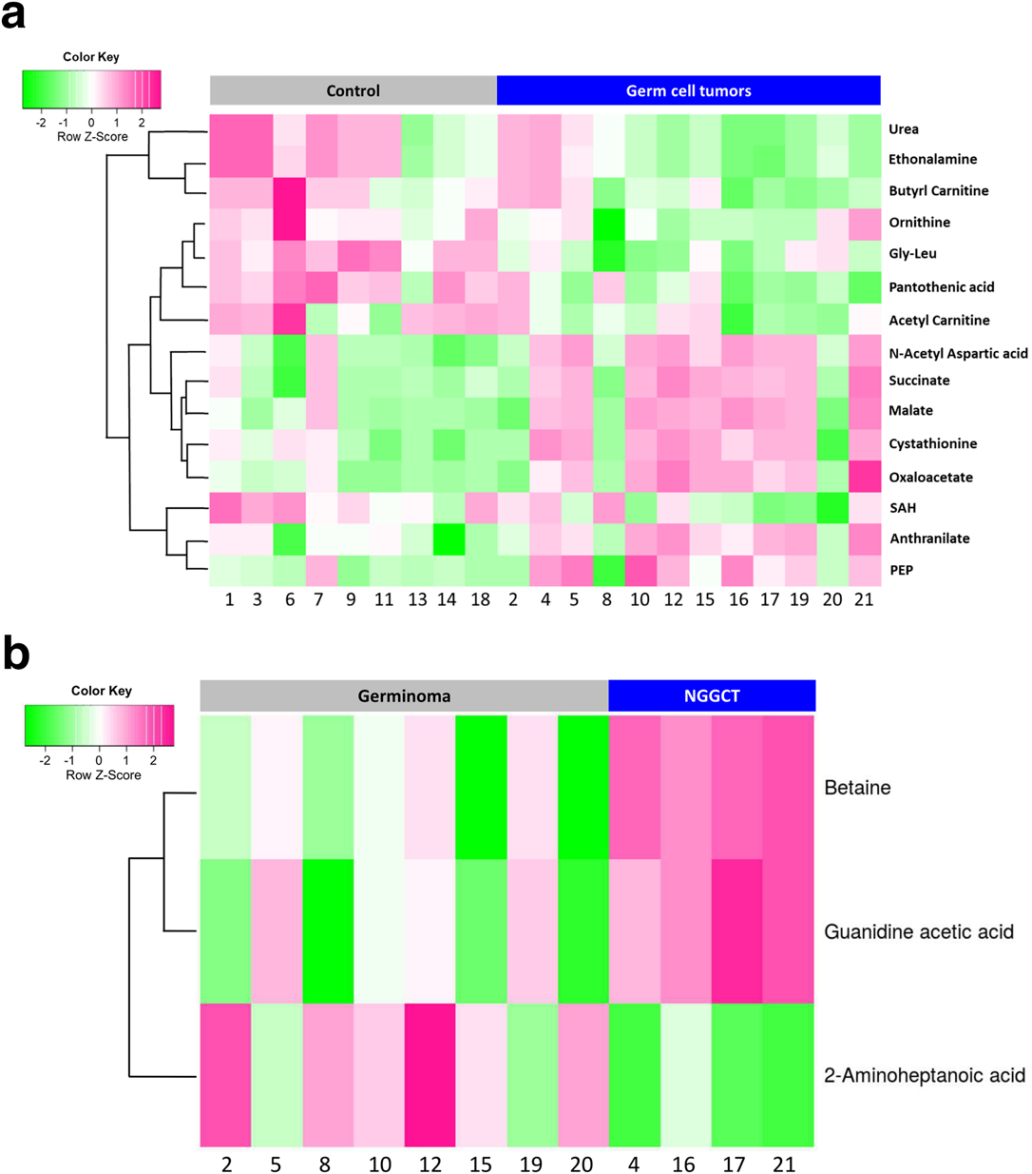
(**A**) Heat map of unsupervised hierarchical clustering of metabolites showing metabolite levels in the CSF of GCT patients and control CSF obtained from patients with no history of cancer. There are 15 differentially expressed metabolites between CSF from GCTs and controls (FDR < 0.25). (**B**) Heat map of unsupervised hierarchical clustering of metabolites showing 3 metabolites (Betaine, Guanidine acetic acid, and 2-aminoheptanoic acid) that are significantly different in the CSF from patients with NGGCTs and germinomas (FDR < 0.3).

Using targeted metabolomics, we detected 146 named metabolites (in ESI positive ionization and negative ionization mode) in the CSF samples. We found differences in the abundance of 15 metabolites between control patients and intracranial GCT patients (FDR < 0.25) (Fig. 2A). Urea and ornithine, urea cycle metabolites, were decreased in GCTs. Tricarboxylic acid (TCA) cycle metabolites, including succinate, malate, and oxaloacetate, were found to be elevated in the CSF from patients with GCTs. In addition, the TCA cycle-related metabolite phosphoenolpyruvate (PEP), increased in the CSF from tumor patients. Pantothenic acid showed a significant decrease in GCTs. Metabolites related to methionine cycle, S-adenosylhomocysteine (SAH) decreased in GCTs, though cystathionine levels were increased. Acetylcarnitine and butyrlcarnitine showed decreased levels in GCTs, whereas N-acetylaspartic acid (NAA) was elevated in a majority of CSF samples from tumor patients.

### CSF metabolites in germinoma versus Non-germinomatous germ cell tumors

**Figure 3:**
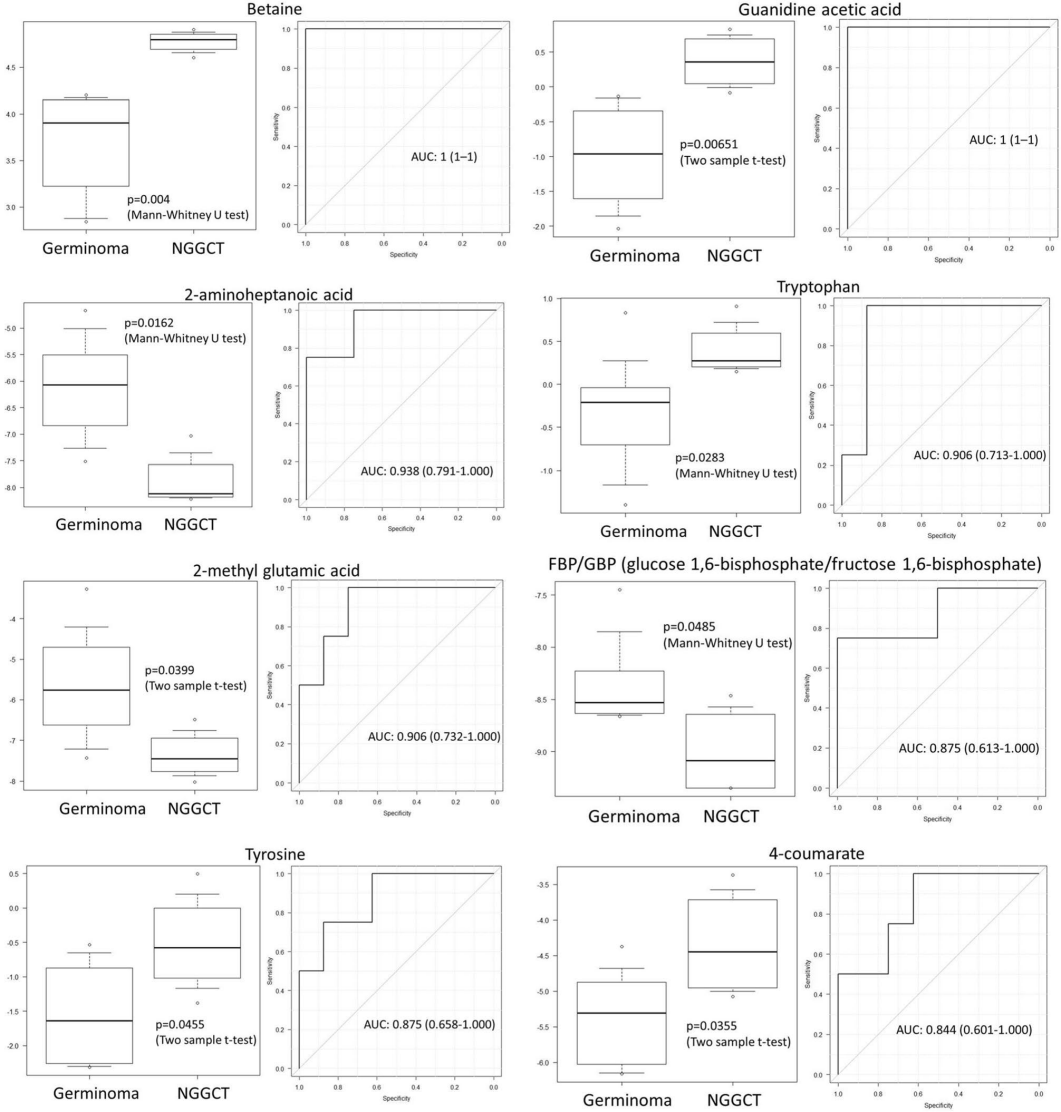
Box plots and area under the ROC curve of 8 metabolites. The levels of these metabolites are significantly different between germinoma and NGGCT. The CSF from patients with NGGCTs have elevated levels of Betaine, guanidine acetic acid, and tryptophan when compared with germinoma patients.

Distinguishing NGGCT form germinoma is important in clinical practice, therefore we compared metabolites in CSF derived from patients with germinoma and NGGCT. We identified three discriminating metabolites with FDR < 0.3 (Fig. 2B). Betaine and guanidine acetic acid were elevated in NGGCTs, and 2-aminoheptanoic acid was decreased in NGGCTs. Moreover, several additional metabolites, such as 4-coumarate, FBP/GBP, 2-methyl glutamic acid, tryptophan, and tyrosine, showed a significant difference between germinomas and NGGCTs (Fig. 3). We investigated the area under the receiver operator characteristics curve (AUC) for each of these differentially abundant metabolites for distinguishing between germinoma and NGGCT. Levels of betaine and guanidine acetic acid had shown AUC = 1, indicating great discriminating power. Six additional metabolites showed AUCs ranging from 0.844 to 0.938 (Fig. 3).

## Discussion

Our study shows that genetic alterations frequently present in GCTs are detectable by analysis of CSF-ctDNA. We detected *KIT* and/or *NRAS* mutations in 3/12 patients (25%). We also identified a *MAP2K1* mutation with a low MAF (case #20). This mutation was not observed in the CSF sample acquired at diagnosis in the same patient (case #8). Therefore, this mutation could have been acquired during tumor progression and/or treatment and might represent tumor evolution. Our validation experiments using CSF samples from patients without CNS tumors indicate that we can confidently identify mutations at a MAF greater than 0.25% (data not shown). Therefore, we favor that the MAP2K1 change is an acquired mutation that represents tumor evolution.

The mutations detected in CSF-ctDNA from patients with GCTs included in our study are consistent with reported mutations in GCTs tissue. Several studies have reported that mutations in MAPK and/or PI3K pathways are the dominant genetic drivers of GCTs^17–19^. A recent study from Japan with 124 CNS GCTs identified mutations in MAPK and/or PI3K pathways such as *KIT* (27.4%), *MTOR* (6.5%), *KRAS* (5.6%), and *NRAS* (3.2%)^17^. The rate of mutation detection in CSF-ctDNA in our study (25% of cases) was lower when compared to the literature showing that ~ 54% of GCTs had mutations in at least one of the genes involved in MAPK or PI3K pathways^17,18^ which are mostly covered by the Oncomine NGS assay. However, it is unclear what mutations were present in the tumors from patients in this study because mutation analysis of tumor tissues was not available.

An average of 1.71 ml of CSF from tumor patients was utilized to extract ctDNA, and the median amount of extracted cell-free DNA (cfDNA) for library preparation was 5.13 ng (Table 1). Considering the recommended cfDNA input for the Oncomine assay is 20 ng, the low volumes of CSF and low amounts of cfDNA might affect the rate of detecting mutations in this study. Our results suggest that volumes larger than 1.7 ml of CSF will be required to increase the sensitivity of CSF-ctDNA analysis in patients with GCTs using the Oncomine assay. However, we successfully detected mutations in case #4, starting with 1.38 ml of CSF volume and 3.14 ng of cfDNA used for library preparation and sequencing, indicating that small cfDNA amounts can provide good results in isolated cases.

Our study has the limitation that we were not able to evaluate some genetic alterations reported in CNS GCTs, such as *NF1*, *CBL*, and *FGD6*, which are not covered by the Oncomine Pan-Cancer Cell-Free Assay. Despite these limitations, our results suggest that evaluation of ctDNA in the CSF of patients with GCTs can be informative and identify mutations that are common drivers of CNS GCTs. Larger CSF volumes and NGS assays specifically designed to interrogate genes known to be altered in GCTs might be required to increase the sensitivity of this method.

We also investigated metabolites in the CSF from GCT patients and found significant differences in the levels of metabolites between GCT and control samples. We observed several altered metabolites related to the TCA cycle including succinate, malate, oxaloacetate, and PEP. Elevated succinate, malate (malic acid), and PEP levels were also demonstrated in the CSF of IDH-mutant glioma patients, in our previous study^14^, suggesting that elevations of these metabolites might be a common finding in the CSF of patients with various types of CNS tumors^20^. SAH and cystathionine levels were decreased and increased in GCTs, respectively. These metabolites are involved in the methionine cycle^21–23^. A recent study reported accumulated cystathionine in tissue samples from 1p/19q co-deleted gliomas, due to deletions of enzyme genes located in chromosome 1p^22^. However, the mechanisms for altered levels of SAH and cystathionine in the CSF of patients with GCTs remain to be elucidated.

Acylcarnitines are essential for the oxidative catabolism of fatty acids. They transport fatty acids into the mitochondria, and fatty acids are oxidized (β-oxidation) and converted to energy through the TCA cycle^24^. A study showed that plasma levels of acetylcarnitine negatively correlated with tumor grade in patients with hepatocellular carcinoma^24^. Studies on plasma metabolites indicate that changes in the MAPK or PI3K pathways are associated with alterations in metabolites including amino acids, acylcarnitines, and phosphatidylcholines^25,26^. Therefore, we assumed that GCTs, often associated with mutations in either MAPK or PI3K pathways, exhibited decreased levels of acetylcarnitine and butyrlcarnitine. However, samples were not available for us to explore this relationship further with tissue-based experiments.

NAA is the second most abundant metabolite in the brain, and it is synthesized from aspartate and acetyl-coenzyme A in neurons, by the enzyme aspartate *N*-acetyltransferase (Asp-NAT)^27^. Therefore, NAA is used as a neuron-specific marker, for instance, lower NAA concentration in CSF have been reported to correlate with worse clinical functioning and lower brain volume in multiple sclerosis patients^28^. We detected increased NAA in the CSF of most GCT patients. However, the interpretation of NAA levels should be done with caution, since NAA levels in CSF can be influenced by age and intracranial pressure^29^. The majority of GCT patients in this study were children or adolescents with non-communicating hydrocephalus with slightly or moderately increased intracranial pressure. In contrast, many of the control CSF samples in our study were elderly patients with NPH. Interestingly, recent studies have shown high expression of NAA metabolism enzymes in brown adipocytes suggesting that that NAA may be involved in lipid synthesis and histone acetylation. Higher expression of NAA synthesizing enzyme and increased amount of NAA, have been associated with worse survival in various cancer types^30^. Elevated NAA was also seen in the CSF from patients with both IDH-mutant and IDH-wildtype gliomas in our previous study^14^.

The levels of several metabolites were significantly different between germinomas and NGGCT. Differentiation between germinomas and NGGCTs is of crucial clinical importance. Increased levels of AFP in serum/CSF indicate that the GCT contains a component of a yolk sac tumor or immature teratoma. Except for germinoma with syncytiotrophoblastic cells, elevated β-HCG suggests an association with choriocarcinoma, embryonal tumor, or immature teratoma. GCTs with a highly malignant component require intensive chemotherapy and corticospinal irradiation^4,5^. Therefore, reliable diagnosis differentiating germinoma from NGGCT is essential to determine optimal patient treatment. Identification of the teratoma/immature teratoma component is also important because these components can cause large cystic masses (growing teratoma syndrome), during treatment^31^. We detected significantly elevated levels of betaine in NGGCT (Figs. 2B and 3). Betaine is known as an important osmotic regulator, has antioxidative or anti-inflammatory activity, and is a methyl group donor. Betaine distributes, particularly, to the kidneys, liver, and brain^23,32^. Although our sample size is small (4 NGGCTs vs. 8 germinomas), our findings indicate that several metabolites have the potential to discriminate germinomas from NGGCT. Therefore, the analysis of CSF metabolites could be instructive in identifying the presence of GCTs with malignant components that should be treated more aggressively.

**Figure 4:**
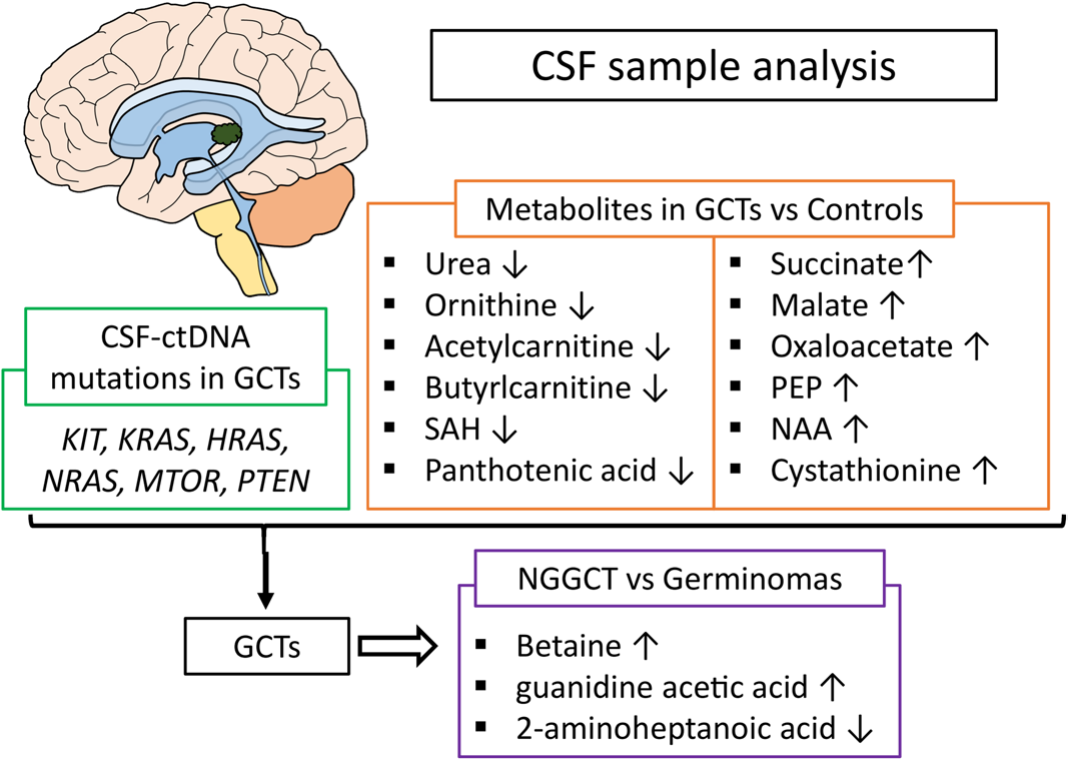
Summary of alterations in CSF ctDNA and metabolites identified in our study. Evaluation of ctDNA and metabolites in CSF has the potential to differentiate patients with GCTs from patients with non-neoplastic diseases and to discriminate patients with NGGCT from patients with germinomas.

In conclusion, our results for the first time suggest that ctDNA and metabolites in CSF can serve as novel biomarkers for CNS GCTs, and can be useful in differentiating germinomas from NGGCTs (Fig. 4). Such biomarkers could also be potentially used to monitor patients following initial treatment and should be further investigated with a multi-institutional prospective study.

## Methods

### Patients

We included intracranial GCT patients treated at Hiroshima University Hospital, Hiroshima, Japan, from 2015 to 2018, or at Memorial Hermann Hospital/University of Texas Health Science Center at Houston, Houston, Texas, from 2018 to 2019. Samples from patients (n = 12) and controls (n = 9) were utilized. The cohort consisted of 8 germinomas, 1 mature teratoma, 1 immature teratoma, and 1 yolk-sac tumor. The control group consisted of 6 normal pressure hydrocephalus (NPH) patients, 1 cytomegalovirus infection, 1 toxoplasmosis, and 1 granulomatous inflammation. CSF was collected during the endoscopic intraventricular intervention (biopsy/ETV) or lumbar puncture (LP) as part of routine clinical practice. Demographic information is summarized in Fig. 1. All CSF samples from GCT patients were obtained before adjuvant chemotherapy and radiotherapy except one patient. A sample from a GCT patient (case #20) was the recurrence of case #8, and we collected the sample after treatment for recurrence. Control patients had no history of cancer.

This study was approved by the institutional review board (IRB), at Hiroshima University Hospital and University of Texas Health Science Center at Houston; respectively, in accordance with the declaration of Helsinki and its later amendments. Informed consent was obtained from all patients.

### CSF collection

CSF samples were collected from the endoscopic intraventricular approach intraoperatively or lumbar puncture as part of routine clinical practice. Samples were processed within 3 h from the collection and centrifuged at 1,000 *g* for 10 min at 4 °C. The cell pellet was discarded, and the supernatant was immediately stored at -80 °C until the time of the analysis.

### ctDNA analysis

A range of 0.38–2.88 mL of thawed CSF supernatant was used for the extraction of ctDNA. We used QIAamp Circulating Nucleic Acid Kit (Qiagen, Germany), following manufacturer instructions. The cell-free DNA (cfDNA) concentration was quantified using the Qubit dsDNA High Sensitivity Assay kit (Thermo Fisher Scientific, USA). For library preparation, we used Oncomine Pan-Cancer Cell-Free Assay (Thermo Fisher Scientific, USA) to detect mutations in CSF-ctDNA as per manufacturer’s instructions. This next-generation sequencing (NGS) panel covers alterations in 52-genes, including hotspot (SNVs) and short indels in *AKT1, ALK, AR, ARAF, BRAF, CHEK2, CTNNB1, DDR2, EGFR, ERBB2, ERBB3, ESR1, FGFR1, FGFR2, FGFR3, FGFR4, FLT3, GNA11, GNAQ, GNAS, HRAS, IDH1, IDH2, KIT, KRAS, MAP2K1, MAP2K2, MET, MTOR, NRAS, NTRK1, NTRK3, PDGFRA, PIK3CA, RAF1, RET, ROS1, SF3B1, SMAD4,* and *SMO*. Although the recommended cfDNA input amount is 20 ng, the majority of our tumor samples did not reach 20 ng, the cfDNA input ranged from 2.25–20 ng. We chose 2 control samples with the highest cfDNA concentration. The final ctDNA libraries were purified using AMPure XP magnetic beads (Beckman Coulter, Germany) and quantified using 4,200 TapeStation (Agilent, USA). Templating and sequencing were performed using the Ion 540 Chip on the Ion Chef and S5 XL systems with tag sequence technology (Thermo Fisher Scientific, USA). NGS data was analyzed with Ion Reporter Software (version 5.10).

### Metabolomic analysis

For metabolomic analysis, 0.12 mL of CSF supernatant from each patient was used for the extraction of metabolites as described previously^33,34^. Targeted metabolic profiling was carried out by LC–MS/MS, 6,495 QQQ Mass spectrometer (Agilent Technologies, Santa Clara, CA) equipped with electro spray ionization (ESI) source. Single Reaction Monitoring (SRM) mode of ions with mass to charge ratio for metabolites were utilized for relative quantitative analysis of metabolites. We measured the metabolites using three different chromatographic methods (see supplementary methods), in each method metabolites were normalized with the spiked internal standards and data were log2-transformed on a per-sample, per-method basis. For clustering analysis, we performed the moderated t-test in the LIMMA package, to compare the differential metabolite levels between two groups^35^. Differentially expressed metabolites were identified after adjusting p-values for multiple hypothesis testing using the Benjamini–Hochberg method^36^ and a false discovery rate (FDR) of < 0.25. Unsupervised clustering was used in this study and the analysis was conducted in R (version 3.5.2). AUC (area under the receiver operating characteristic curve) and its 95% confidence interval were evaluated by the “DeLong” method with EZR (version 1.40)^37^. For the box plot, differences in metabolites between two groups of patients were analyzed using the Mann–Whitney U test or two-sample t-test, depending on whether the Kolmogorov–Smirnov test showed a significant difference from the normal distribution in each data.

## Supplementary information

Supplementary information

## Data Availability

All data generated or analyzed during this study are included in this published article.

## Abbreviations

GCT: Germ cell tumors
CNS: Central nervous system
NGGCTs: Non-germinomatous GCTs
CSF: Cerebrospinal fluid
AFP: α-Fetoprotein
β-HCG: β-Subunit of human chorionic gonadotropin
PLAP: Placental alkaline phosphatase
ETV: Endoscopic third ventriculostomy
ctDNA: Circulating tumor DNA
NPH: Normal pressure hydrocephalus
LP: Lumbar puncture
cfDNA: Cell-free DNA
NGS: Next-generation sequencing
ESI: Electro spray ionization
SRM: Single reaction monitoring
FDR: False discovery rate
MAF: Mutant allele fraction
TCA: Tricarboxylic acid
PEP: Phosphoenolpyruvate
SAH: S-adenosylhomocysteine
NAA: N-acetylaspartic acid
Asp-NAT: Aspartate *N*-acetyltransferase
