# Supplementary material for: Cerebrospinal fluid ctDNA and metabolites are informative biomarkers for the evaluation of CNS germ cell tumors
Source: Sci Rep. 2020 Aug 31;10:14326. doi: 10.1038/s41598-020-71161-0 (PMC7459305; doi:10.1038/s41598-020-71161-0)
Supplement: Supplementary file 1 — Supplementary information [file 41598_2020_71161_MOESM1_ESM.docx]

**Cerebrospinal Fluid ctDNA and Metabolites are Informative Biomarkers for the Evaluation of CNS Germ Cell Tumors**

Takeshi Takayasu^1,2^, Mauli Shah^1^, Antonio Dono^3^, Yuanqing Yan^3^, Roshan Borkar^4^, Nagireddy Putluri^4^, Jay-Jiguang Zhu^3,5^, Seiji Hama^2^, Fumiyuki Yamasaki^2*^, Hidetoshi Tahara^6^, Kazuhiko Sugiyama^7^, Kaoru Kurisu^2^, Yoshua Esquenazi^3,5,8^, Leomar Y. Ballester^1,3,5*^

^1^Department of Pathology and Laboratory Medicine, the University of Texas Health Science Center at Houston, Houston, Texas, USA

^2^Department of Neurosurgery, Graduate School of Biomedical and Health Sciences, Hiroshima University, Hiroshima, Japan

^3^Vivian L. Smith Department of Neurosurgery, the University of Texas Health Science Center at Houston, UTHealth McGovern Medical School, Houston, Texas, USA

^4^Metabolomics Core, Alkek Center for Molecular Discovery, Baylor College of Medicine, Houston, Texas, USA

^5^Memorial Hermann Hospital-TMC, Houston, Texas, USA

^6^Department of Cellular and Molecular Biology, Graduate School of Biomedical and Health Sciences, Hiroshima University, Hiroshima, Japan

^7^Department of Clinical Oncology & Neuro-oncology Program, Hiroshima University Hospital, Hiroshima, Hiroshima, Japan

^8^ Center for Precision Health, School of Biomedical Informatics, The University of Texas Health Science Center at Houston.

**e-mail address:** Takeshi Takayasu: ttakayasu-nsu@umin.ac.jp, Mauli Shah: Mauli.H.Shah@uth.tmc.edu, Antonio Dono: Antonio.Dono@uth.tmc.edu, Yuanqing Yan: Yuanqing.Yan@uth.tmc.edu, Roshan Borkar: roshanudps@gmail.com, Nagireddy Putluri: putluri@bcm.edu, Jay-Jiguang Zhu: Jay.Jiguang.Zhu@uth.tmc.edu, Seiji Hama: shama@hiroshima-u.ac.jp, Fumiyuki Yamasaki: fyama@hiroshima-u.ac.jp, Hidetoshi Tahara: toshi@hiroshima-u.ac.jp, Kazuhiko Sugiyama: brain@hiroshima-u.ac.jp, Kaoru Kurisu: kuka422@hiroshima-u.ac.jp, Yoshua Esquenazi: Yoshua.EsquenaziLevy@uth.tmc.edu, Leomar Y. Ballester: Leomar.Y.Ballester@uth.tmc.edu

***Corresponding Author:**

Leomar Y. Ballester, M.D., Ph.D.

Assistant Professor

Molecular Genetic Pathology and Neuropathology, Department of Pathology and Laboratory Medicine, University of Texas Health Science Center at Houston

6431 Fannin St., MSB 2.136, Houston, TX 77030

Tel.: 713-500-5336, Leomar.Y.Ballester@uth.tmc.edu

Fumiyuki Yamasaki, M.D., Ph.D.

Associate Professor

Department of Neurosurgery, Graduate School of Biomedical and Health Sciences

Hiroshima University, 1-2-3, Kasumi, Minami-ward, Hiroshima City, Hiroshima, 734-8551, Japan

Tel.:+81-082-257-5227, [fyama@hiroshima-u.ac.jp](mailto:fyama@hiroshima-u.ac.jp)

**Supplementary methods**

Metabolic analysis of CSF samples: Metabolites were extracted from CSF, and CSF pool was used as quality controls following the extraction procedure. All the CSF samples used for this study were stored at -140oC. 100 ul of CSF was used for the metabolic extraction. The extraction step started with the addition of 750 µL ice-cold methanol:water (4:1) containing 20 µL spiked internal standards to each CSF sample. Ice-cold chloroform and water was added in a 3:1 ratio for a final proportion of 1:4:3:1 water:methanol:chloroform:water. The organic (methanol and chloroform) and aqueous layers were mixed, dried and resuspended with 50:50 methanol: water. The extract was deproteinized using a 3kDa molecular filter (Amicon ultracel-3K Membrane; Millipore Corporation, Billerica, MA) and the filtrate was dried under vacuum (Genevac EZ-2plus; Gardiner, Stone Ridge, NY). Prior to mass spectrometry, the dried extracts were re-suspended in identical volumes of injection solvent composed of 1:1 water: methanol and were subjected to liquid chromatographymass spectrometry.

**Liquid Chromatography-Mass spectrometry (LC-MS**)

Extracted CSF samples were injected and analyzed using a 6490 triple quadrupole mass spectrometer (Agilent Technologies, Santa Clara, CA) coupled to a HPLC system (Agilent Technologies, Santa Clara, CA) via single reaction monitoring (SRM). A total number of ~129 endogenous metabolites were chosen due to their involvement in central pathways and in a number of other biological functions. Source parameters were as follows: Gas temperature- 250 °C; Gas flow- 14 l/min; Nebulizer - 20psi; Sheath gas temperature - 350 °C; Sheath gas flow- 12 l/min; Capillary - 3000 V positive and 3000 V negative; Nozzle voltage- 1500 V positive and 1500 V negative. Approximately 8–11 data points were acquired per detected metabolite. We used different methods to measure the ~129 metabolites as follows:

**Method A:** ESI positive mode was used in method A. The HPLC column was Waters XBridge Amide 3.5 µm, 4.6 x 100 mm. Mobile phase A and B were 0.1% formic acid in water and acetonitrile respectively. Gradient: 0 min-85 % B; 3-12 min- 85% to 10 % B, 12-15 min-10 % B, 16 min- 85% -B, followed by re-equilibration end of the gradient- the 23 min to the initial starting condition 85% B. Flow rate: 0.3 ml/min. Injection Volume 5ul.

**Method B:** ESI negative mode was used in method B. The HPLC column was Waters XBridge Amide 3.5 µm, 4.6 x 100 mm. Mobile phase A and B were 20 mM ammonium acetate in water with pH 9.0 and 100% acetonitrile respectively. Gradient: 0 min-85 % B; 0-3 min- 85 % to 30% B, 3-12 min-30%-2 % B, 12-15 min- 2% -B, 15-16 min- 85% B followed by re-equilibration end of the gradient- the 23rd min to the initial starting condition 85% B. Flow rate: 0.3 ml/min. Injection Volume 10ul.

**Method C:** ESI negative mode was used in method C. The HPLC column was Luna 3 µm NH2 100 A, 150 x 2 mm. Mobile phase A and B were 20 mM ammonium acetate in water with pH 9.0 and 100% acetonitrile respectively. Gradient: 0 min-85 % B; 0-3 min- 85 % to 30% B, 3-12 min-30%-2 % B, 12-15 min- 2% -B, 15-16 min- 85% B followed by re-equilibration end of the gradient- the 23rd min to the initial starting condition 85% B. Flow rate: 0.3 ml/min. Injection Volume 10ul.
